# Effect of double- density foot orthoses on ground reaction forces and lower limb muscle activities during running in adults with and without pronated feet

**DOI:** 10.1186/s13102-025-01095-5

**Published:** 2025-03-21

**Authors:** Ebrahim Piri, Vahid Sobhani, AmirAli Jafarnezhadgero, Ehsan Arabzadeh, Alireza Shamsoddini, Matteo Zago, Urs Granacher

**Affiliations:** 1 Department of Sports Biomechanics, Faculty of Educational Sciences and Psychology, University of Mohaghegh Ardabili, Ardabil, Iran; 2 Department of Biomedical Sciences for Health, Università degli Studi di Milano, Milano, Italy; 3 Department of Sport and Sport Science, Exercise and Human Movement Science, University of Freiburg, Freiburg, Germany; 4 Exercise Physiology Research Center, Life Style Institute, Baqiyatallah University of Medical Sciences, Tehran, Iran; 5 Student Research Committee, Baqiyatallah University of Medical Sciences, Tehran, Iran

**Keywords:** Flat feet, Insoles, Electromyography, Kinetics, Gait analysis

## Abstract

**Background:**

The analysis of ground reaction forces and muscle activities during walking or running can help clinicians decide on the usage of foot orthoses, particularly in individuals with pronated feet. Here, we aimed to investigate the effects of double- density foot orthoses on running kinetics and lower limb muscle activities in adults with and without pronated feet.

**Methods:**

Forty male adults with pronated feet (PF: *n* = 20, age = 25.4 ± 0.3 years, body-mass-index [BMI] = 23.3 ± 1.2 kg/m^2^) and without pronated feet (WPF: *n* = 20, age = 26.4 ± 1.0 years, BMI = 24.0 ± 0.7 kg/m^2^) volunteered to participate in this study. The study was registered with the Iranian Registry of Clinical Trials (IRCT20220129053865N1). Ground reaction forces (F_x_, F_y_, F_z_) and lower limb muscle activities (e.g., m. gastrocnemius) were recorded using surface electromyography (EMGs) during running at a constant speed of 3.2 m/s over an 18-m walkway with an embedded force plate. EMGs were normalized to maximum voluntary isometric contractions.

**Results:**

Test-retest reliability for running speed data was excellent for PF and WPF groups and for the entire study cohort with intraclass correlation coefficients > 0.95. The 2-way ANOVA revealed lower peak F_z_ (*p* = 0.011; d = 1.226), lower time-to-peak for F_x_ (*p* = 0.023, d = 1.068), F_y_ (*p* = 0.025, d = 1.056), and F_z_ (*p* = 0.045, d = 0.931) during running with foot orthoses in PF individuals. During the loading phase, PF and WPF exhibited lower gastrocnemius (WPF: *p* = 0.005, d = 1.608; PF: *p* = 0.001, d = 2.430 ) and vastus medialis (WPF: *p* < 0.001, d = 2.532; PF: *p* < 0.001, d = 2.503) activity when running with foot orthoses.

**Conclusions:**

Although double- density foot orthoses resulted in some beneficial biomechanical effects such as lower muscle activation (e.g., m. vastus medialis) in individuals with PF, foot orthoses constructions need further modifications to achieve even better running mechanics to enhance performance and lower limbinjury occurrence.

**Trial registration:**

IRCT20220129053865N1 (Registration date 19/08/2024).

**Supplementary Information:**

The online version contains supplementary material available at 10.1186/s13102-025-01095-5.

## Background

Running has gained in popularity as recreational and competitive activity due to the minimal equipment needed and the time efficient training, making it an highly effective exercise regime for performance development and health promotion [1]. Data on running-related injuries (RRIs) vary between 2.5 and 38 injuries per 1000 h of running [2–4].

The aetiology of RRIs comprises greater peak hip adduction and vertical loading rates during running, and lower limb malalignment such as pronated feet (PF) [5].

During running, peak vertical ground reaction forces (GRF) are typically in the range of ~ 1.5-3 time body mass [6], whereas peak forces on the distal end of the tibia are typically 6–14 time body mass [7, 8]. Increases in GRF metrics are routinely assumed to reflect increases in internal structure loading such as tibial bone loading [8]. Thus, the relative damage due to impact forces appears to be small regardless of the magnitude of the respective impact force (e.g., 1.8 vs. 1.55 body mass) [9]. Loading rate, calculated from the slope of the vertical GRF over a specified time period, is a common parameter used to evaluate RRIs [10]. The derived loading rate value represents the rate at which the acceleration of the whole body center of mass changes immediately following initial ground contact [10]. Faster time-to-peak (TTP) in vertical GRF indicates a rapid loading rate, often associated with heel-strike running or high-impact landings [11]. Medio-lateral forces during running are critical for maintaining balance, stability, and efficient and economic movement [12]. In general, the peak medial GRF during the push-off phase is typically small relative to the vertical GRF and is mostly caused by the body’s need to stabilize lateral motion and manage body segment alignment. The medial force tends to be greatest during the late stance phase (just before the toe-off). Authors from studies examining running biomechanics have reported that the medial force is often less than 10% of the peak vertical GRF [13]. The braking force during the heel contact is typically the largest negative force observed during the running cycle. The magnitude of the braking force is in the range of 5–15% of the individual’s body mass [14]. Free moment is a measure of the torsional force (moment) around the vertical axis at the point of contact between the foot and the ground [15]. The free moment represents the rotational interaction between the ground and the foot caused by transverse-plane forces and torques. The parameter free moment is influenced by factors such as the foot strike pattern, tibial rotation, and the alignment of the body relative to the GRF vector [16]. There is preliminary evidence that PF may raise the torsional load and alter the free moment, which in turn has frequently been used as torsional lower limbs stress index [16, 17]. Historically, it has been argued that pathological foot mechanics may be associated with lower extremity malalignment [18]. In fact, there is evidence indicating that rearfoot motion (eversion) closely corresponds with tibial motion (internal rotation) [19, 20] and is potentially associated with transverse plane rotations at the hip [21]. This model of lower extremity joint coupling implies a theoretical link between foot pronation and lower limb alignment including overuse injuries of the lower limbs, medial tibial stress syndrome and patellofemoral pain [22]. Therefore, runners should be regularly monitored to identify those with PF. Subsequently, PF runners could receive passive (i.e., orthoses) and/or active treatment (i.e., exercise). In terms of passive treatment, foot orthoses (FOs) are often used as a conservative approach to improve running mechanics (e.g., lower peak rear foot eversion) in individuals with PF [23].

Previously, researchers have summarized the effects of FOs on running economy and performance as well as muscle activity in the form of systematic literature reviews [24–26]. Authors from these studies primarily focused their literature analysis on the impact of footwear-construction on running kinetics and kinematics [24–26]. Notably, their findings demonstrated that running economy was reduced with shock-absorbing FOs and increased with carbon fiber FOs that are characterized by distinct longitudinal bending stiffness. Additionally, FO application resulted in increased lower limbs muscle activation while running [25]. Previously it has been postulated that the most widely applied kinetic outcomes are loading rate and impact force [27]. However, the effects of FOs on these variables are controversially reported in the literature and therefore remain unresolved [27]. More recently, findings from a systematic review with meta-analysis demonstrated that both, custom and off-the-shelf arch-support FOs reduced peak plantar pressure at the medial heel, lateral heel, and medial forefoot, but increased plantar pressure at the mid-foot [26]. A reduction in initial ankle inversion was found when a raised heel cup was integrated with arch-support FOs [26]. A medial post integrated with arch support exhibited a reduced ankle and tibial range of motion. Custom FOs, however, unfavorably affected running economy and perceived exertion of recreational runners [26]. Overall, findings from the meta-analysis of Jor et al. [26] indicate that although FOs have a few beneficial biomechanical lower limb effects in healthy populations, FO constructions should be modified and adapted to achieve better running performance and prevent injury occurrence.

**Fig. 1:**
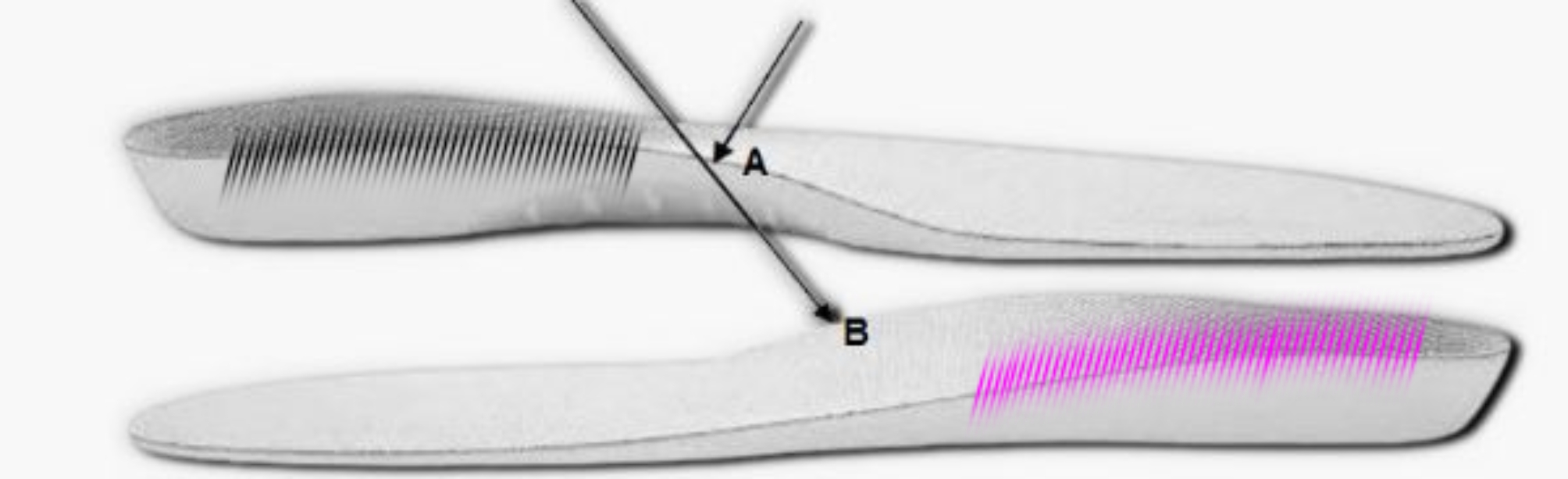
Double density FO design. **(A)** The longitudinal arc medial part was elevated as much as 12 mm; **(B)** The stiffness of the medial part was increased up to 60 shore A (pink area [with the dimension of half area of FO rear foot section]), and 30 shore A in the lateral part (black area)

FOs designed for PF individuals aim to restore normal foot dynamic function during sports-related activities such as running [28]. These corrective foot alignment effects are usually obtained by means of proper configurations of the respective orthotic components [23]. Therefore, understanding the effects of FO component features on running mechanics is essential to effectively design FOs. Medial wedge FOs are the most commonly used orthoses to alter lower limb biomechanics during walking or running in individuals with PF [29]. Rearfoot posting facilitates foot orthoses use, as forefoot posting is challenging to fit within the shoe and may cause discomfort [30]. Accordingly, double- density FOs have been developed which are equipped with low density in the lateral part and high density in the medial part (Fig. 1). Double- density FOs may have a similar effect as motion control footwear as they both effectively reduce [31] the magnitude of foot pronation during running and the risk of sustaining running-related injuries [32]. Currently, it is unresolved whether double- density FOs have positive effects on running mechanics, more specifically peak GRFs and their time-to-peak, free moments, and muscle activities in adults with PF [29, 33, 34].

To the best of our knowledge, there are no studies available that examined GRFs and muscle activities during running in adults with PF compared to individuals with normal foot posture while using double-density FOs. Therefore, we aimed to investigate the effects of double- density FOs on running kinetics and lower limb muscle activities while running at a constant speed in adults with and without PF. We expect that double- density FOs reduce both, GRF amplitudes and muscular activities, particularly in adults with PF [29, 33, 34].

## Methods

### Participants

Forty male adults with and without PF volunteered to participate in this study (Table 1). The G*Power software was used to calculate an a priori power analysis with the F test family using a related study that evaluated the vertical force component in PF runners [35]. An alpha level of 0.05, a type II error rate of 0.20 (80% statistical power), and an effect size *f* of 0.23 were set to compute the power analysis. Findings showed that 40 participants (20 per group) would be needed to achieve a significant group-by-condition interaction effects. Table 1 contains a summary of the inclusion and exclusion criteria for study participation. Demographic and anthropometric characteristics of the participants are shown in Table 2. All participants were right-footed based on the results of a ball-kicking test. This study adheres to CONSORT guidelines ( Appendices 1 and 2).

**Table 1:** Inclusion and exclusion criteria for study participation

Groups	Inclusion criteria	Exclusion criteria
PF group	Male	Females. Irrespective of sex, a history of musculoskeletal surgery in the trunk and/or lower limbs was defined as exclusion criteria. To be included in this study participants had no Surgical treatment of the lower limbs or trunk two years prior to the start of the study were defined as exclusion criteria.
	BMI < 25 kg/m^2^	Acute neuromuscular or orthopedic disorders (except for PF)
	Rearfoot eversion angle > 4° [36, 37]	Lower limb length asymmetry exceeding 5 mm
	Navicular drop > 10 mm	Engagement in strenuous physical activity within 48 h before testing
	A foot posture index > 10 [38]	Midfoot or forefoot strike pattern
	Rearfoot striker	
WPF group	Male	History of musculoskeletal surgery in the trunk and/or lower limbs. To be included in this study participants had no surgical treatment at the lower limbs or trunk two years prior to the start of the study.
	BMI < 25 kg/m^2^	Acute neuromuscular or orthopedic disorders
	Rearfoot eversion angle < 4° [36, 37]	Lower limb length asymmetry exceeding 5 mm
	5 < Navicular drop < 10 mm	Engagement in strenuous physical activity within 48 h before testing
	6 < A foot posture index < 10 [38]	Midfoot or forefoot strike pattern
	Rearfoot striker	Forefoot striker

Notes: WPF, without pronated feet; PF, pronated feet; BMI, body-mass-index

**Table 2:** Demographic and anthropometric characteristics of the study participants.

Variables	WPF group	PF group	*p*-values
Age (years)	25.4 ± 0.3	26.4 ± 1.0	0.091
Body height (cm)	177.3 ± 7.2	181.7 ± 6.4	0.883
Body mass (kg)	73.2 ± 14.8	79.6 ± 13.2	0.407
BMI (kg/m^2^)	23.3 ± 1.2	24.0 ± 0.7	0.501
Navicular drop (mm)	7.5 ± 1.2	13.4 ± 1.5	< 0.001*
Foot posture index	3.2 ± 0.5	7.8 ± 0.9	< 0.001*

Notes: WPF, without pronated feet; PF, pronated feet; BMI, body-mass-index. Significant results were denoted in bold

The applied foot posture index [38, 39] included six items including palpation of the talus head, curvature above and below the lateral malleolus, calcaneus position in the frontal plane, prominence of the malleolus, congruence of the medial longitudinal arch, and forefoot abduction/adduction. A podiatrist with ~ 11 years of experience applied the foot posture index evaluation. Each item was rated on a visual analogue scale from − 2 to + 2, yielding a total score range of -12 to + 12. Negative values indicate a supinated foot posture, while positive values indicate PF posture, with scores of 6–10 classified as PF [38, 39]. Validity of the foot posture index has been reported in a previous study [38]. The foot posture index predicted about 64% of the variance of valgus index values in monopedal static standing condition and showed good inter item reliability (Cronbach’s α = 0.83) [38].

All eligible participants provided written informed consent prior to the commencement of the study. Ethics approval was received from the ethical committee of Baqiyatallah Medical Sciences University, Iran (IR.BMSU.BAQ.REC.1403.066). The study procedures were in accordance with the latest version of the Declaration of Helsinki. The study was registered with the Iranian Registry of Clinical Trials (IRCT20220129053865N1).

### Equipment and procedures

Before the study started, participants were familiarized with the applied tests and the test set-up including the 18-m walkway with an embedded force plate (Bertec Corporation, Columbus, OH, USA). Participants received an individualized double-density FO matched to their foot size. This approach was uniformly applied for the PF and the normal feet groups. The FOs were made of ethylene-vinyl acetate (EVA) with a medial stiffness of Shore 60, a lateral stiffness of Shore 30, and a standardized medial longitudinal arch support height.

### Overground running

Laboratory sessions were consistently scheduled between 10:00 and 12:00 AM. Prior to testing, participants completed a standardized 10-minute warm-up protocol, which included jogging at low-to-moderate intensities for 7 min followed by 3 min of dynamic stretching. For the running trials, an 18-m walkway with an embedded force plate at the midsection of the lane was used to collect GRF data at a sampling rate of 1000 Hz. The force plate, measuring 60 cm in length and 40 cm in width, was aligned lengthwise with the running direction. All participants were familiarized with the laboratory setup and instructed to run across the walkway at a constant speed of 3.2 m/s. To be eligible for further data analysis, variation within running trials had to be in a 5% time range. Two sets of infrared photocells (South Wales, Australia) were used to control and monitor running speed. Six test trials were conducted per condition, with each participant receiving three familiarization trials to ensure consistent speed and proper foot contact with the force plate using their dominant foot. Test–re-test reliability for running speed data demonstrated ICC > 0.95 for each group and in total and can be considered excellent.

Trials were discarded based on the following criteria: (i) the dominant foot did not touch the force plate; (ii) the participant lost balance during the trial.

### Running kinetics

Kinetic data were processed according to previously explained procedures [40]. In brief, GRF data were low-pass filtered at 20 Hz (4th order Butterworth filter, zero lag). The heel strike and toe-off events were identified using the force platforms' data with a 10-N force onset threshold. As dependent variables, GRF during running (F_x_, _y_, _z_), time-to-peak for GRF, and free moments were recorded. These kinetic parameters have proven to be clinically relevant because it has been shown in previous work that they are related to pathological gait/running patterns [41]. More specifically, we extracted the first peak of vertical force (F_zHC_) from GRF data [40]. Then, the anterior (F_yPO_) and posterior (F_yHC_) peak of GRF and the positive (lateral) peak (F_xHC_) and the negative (medial) peak (F_xPO_) from the medial–lateral curve were obtained. GRF values were normalized to body weight (BW) and reported as %BW. Time-to-peak was defined as the interval between initial heel contact and the peak of the vertical GRF component.

The free moment at the foot level was obtained as follows:

$$\:\text{F}\text{M}=\text{M}\text{z}+\left(\text{F}\text{x}\times\:\text{C}\text{O}\text{P}\text{y}\right)-(\text{F}\text{y}\times\:\text{C}\text{O}\text{P}\text{x})$$

where M_z_ represents the moment around the vertical axis, while x and y denote the horizontal components of the center of pressure (COP), and F_x_ and F_y_ are the horizontal GRF components. Additionally, FM amplitudes were normalized relative to BW × height. All running variables were averaged across six trials.

### Surface electromyography

A wireless EMG system (Biometrics Ltd., Nine Mile Point Ind. Est, Newport, United Kingdom) with eight pairs of bipolar Ag/AgCl surface electrodes was used to assess m. tibialis anterior (TA), m. gastrocnemius medialis (Gas-M), m. biceps femoris (BF), m. semitendinosus (ST), m. vastus lateralis (VL), m. vastus medialis (VM), m. rectus femoris (RF), and m. gluteus medius (Glut-M) activities of the dominant limb [42]. We followed the European recommendations for surface EMG (SENIAM) and kept the center-to-center electrode distance at 25 mm [43]. Input impedance and common mode rejection ratio was set at 100 MΩ and > 110 dB, respectively. The skin over the respective muscle bellies was shaved and cleaned with 70% ethanol (C₂H₅OH). The skin was then gently abraded prior to electrode placement. Surface electrodes were positioned on the muscle belly, aligned longitudinally with the muscle fibers [42]. Participants completed two familiarization trials before recording the actual tests. The raw EMG signals were digitized at 1000 Hz, and GRF and EMG data were synchronized using Nexus software (Oxford Metrics, Oxford, United Kingdom). EMG data were processed according to a previous study [42], and root mean square (RMS) values were calculated across all phases. Briefly, test trials were divided into loading (0–15% of the running cycle), mid-stance (15–25% of the running cycle), and push-off (25–40% of the running cycle) phases (Dugan & Bhat, 2005). Maximum voluntary isometric contraction (MVIC) was assessed using a handheld dynamometer to normalize EMG during running relative to MVIC. For each participant, six running trials were recorded under each condition (with and without FOs). A trial was deemed successful if the foot landed centrally on the force plate and if EMG signals were free from artifacts, as confirmed by visual inspection of the live display. MVIC tests were conducted after the running trials for each muscle separately to normalize EMG data. For normalization, the peak RMS values assessed during the running trials were divided by the peak MVIC values and multiplied by 100, with muscle activity reported as %MVIC.

### Statistical analyses

Normality of data distribution was assessed and confirmed using the Shapiro–Wilk test. Accordingly, data are presented as means and standard deviations (SD). A two-way repeated-measures ANOVA was computed with the factors group (PF, WPF individuals) and condition (with and without FOs). Pair-wise comparisons were calculated in case main effect of group orcondition reached the level of statistical significance. Post-hoc tests were calculated using Bonferroni adjusted paired sample t-tests in case “group-by-condition” interactions reached the level of statistical significance.

Effect sizes in the form of partial eta-squared were taken from ANOVA output and transformed into Cohen’s d [44]. Within-group effect sizes were computed using the following equation: mean difference of pre and posttests/pooled standard deviation. According to Cohen [45], d < 0.50 indicate small effects, 0.50 < d < 0.80 indicate medium effects, and d ≥ 0.80 indicate large effects. The significance level was set at *p* < 0.05. Statistical analysis was conducted with SPSS (Version 26).

## Results

### Running kinetics

Significant differences on the “group” factor were found for F_yPO_ (*p* = 0.041; d = 0.950), peak negative free moment (*p* = 0.001; d = 2.379), time-to-peak for F_xPO_ (*p* = 0.005; d = 1.372), time-to-peak for F_yHC_ (*p* = 0.001; d = 1.719), and time-to-peak for F_zHC_ (*p* = 0.012; d = 1.198). Pair-wise comparisons demonstrated lower peak negative free moments and greater F_yPO_, time-to-peak for F_xPO_, time-to-peak for F_yHC_, and time-to-peak for F_zHC_ in the PF compared with the WPF group (Table 3).

Significant main effects for “condition” were observed for F_zHC_ (*p* = 0.011; d = 1.226), and time-to-peak for F_xPO_ (*p* = 0.005; d = 1.366). Pair-wise comparisons demonstrated lower F_zHC_, time-to-peak for F_xPO_, and time-to-peak for F_zHC_ during running with compared to without FOs. Significant “group-by-condition” interactions were detected for time-to-peak for F_xPO_ (*p* = 0.023; d = 1.068), time-to-peak for F_yHC_ (*p* = 0.025; d = 1.056), and time-to-peak for F_zHC_ (*p* = 0.045; d = 0.931) during the loading phase. The post hoc analyses demonstrated lower time-to-peak for F_xPO_, time-to-peak for F_yHC_ and time-to-peak for F_zHC_ in the PF group (but not in the WPF group) during running with compared to without FOs (Table 3).

**Table 3:** Data are means and standard deviations for ground reaction forces (GRFs) and their time-to-peak, during running in individuals with and without PF

Ground reaction forces	WPF group				PF group				Main effect of group (eta squared)	Main effect of condition (eta squared)	Group-by-condition interaction (eta squared)
	Pre	Post	∆%	95%CI	Pre	Post	∆%	95%CI			
F_xHC_ (% BW)	10.18 ± 7.38	6.96 ± 2.85	-31.63	-2.46, 8.89	7.82 ± 2.93	5.41 ± 1.42	-30.81	0.06, 4.75	0.093 (0.770)	0.064 (0.853)	0.782 (0.127)
F_xPO_ (% BW)	-11.12 ± 4.47	-10.3 ± 3.27	-7.37	-3.81, 2.15	-14.53 ± 3.94	-10.87 ± 3.35	-25.18	-7.81, 0.5	0.086 (0.787)	0.062 (0.860)	0.228 (0.540)
F_yHC_ (% BW)	-33.12 ± 13.11	-27.27 ± 10.19	-17.66	-17.2, 5.480	-23.58 ± 8.49	-26.04 ± 6.32	10.43	-4.23, 9.15	0.069 (0.837)	0.584 (0.247)	0.188 (0.594)
F_yPO_ (% BW)	21.67 ± 8.98	18.55 ± 5.59	-14.39	-5.39, 11.62	23.27 ± 6.62	23.43 ± 5.08	0.68	-6.25, 5.92	0.041 (0.950)*	0.546 (0.271)	0.503 (0.300)
F_zHC_ (% BW)	245.4 ± 68.06	191.34 ± 46.82	-22.02	2.18, 105.92	251.46 ± 53.32	220.2 ± 36.25	-12.43	-10.05, 72.57	0.285 (0.478)	0.011 (1.226)*	0.461 (0.327)
Positive FM %(BW*height) × 10^-3^	0.36 ± 0.22	0.31 ± 0.14	-13.88	-0.12, 0.22	0.71 ± 0.25	0.48 ± 0.15	-32.29	-0.02, 0.47	0.405 (0.369)	0.128 (0.692)	0.557 (0.263)
Negative FM %(BW*height) × 10^-3^	-0.40 ± 0.25	-0.28 ± 0.14	-30	-0.34, 0.08	-0.33 ± 0.19	-0.27 ± 0.1	-18.18	-0.18, 0.06	**0.001 (2.293)**	0.056 (0.886)	0.207 (0.569)
TTPF_xHC_ (ms)	29.36 ± 8.95	31.8 ± 7.66	8.31	-9.77, 4.89	29.21 ± 8.58	29.27 ± 4.61	0.20	-7.28, 7.16	0.549 (0.263)	0.597 (0.238)	0.615 (0.220)
TTPF_xPO_ (ms)	103.22 ± 43.23	94.97 ± 43.72	-7.99	-35.74, 52.24	163.57 ± 29.9	96.09 ± 27.94	-41.25	39.07, 95.89	0.005 (1.372)*	0.005 (1.366)*	0.023 (1.068)*
TTPF_yHC_ (ms)	56.08 ± 25.04	73.02 ± 10.11	30.20	-37.12, 3.23	83.48 ± 9.85	75.75 ± 9.02	-9.25	-0.4, 15.85	0.001 (1.719)*	0.377 (0.392)	0.025 (1.056)*
TTPF_yPO_ (ms)	232.24 ± 28.95	225.13 ± 22.09	-3.06	-24.03, 38.25	243.69 ± 11.90	220.54 ± 18.73	-9.49	6.15, 40.14	0.350 (0.419)	0.081 (0.800)	0.342 (0.424)
TTPF_zHC_ (ms)	33.22 ± 10.15	31.38 ± 10.12	-5.53	-8.77, 12.44	46.9 ± 7.93	33.9 ± 14.63	-27.71	5.13, 20.86	0.012 (1.198)*	0.153 (0.648)	0.045 (0.931)*

Notes: WPF, without pronatd feet; PF, pronated feet; BW, body weight; x, medio-lateral direction; y, anterior-posterior direction; z, vertical direction; F_zHC_; peak vertical ground reaction force during heel contact; F_zPO_; peak vertical ground reaction force during the push-off phase; F_yHC_, braking force; F_yPO_, propulsion force; F_xHC_, peak lateral ground reaction force during heel contact; F_xPO_, peak medial ground reaction force during the push-off phase; FM, free moment; TTP, time-to-peak; 95% CI, confidence interval refers to the confidence interval of the difference between with and without FO. Significant results were denoted in bold

### Muscle activities

The statistical analysis showed significant main effects of “group” for TA (*p* < 0.001; d = 1.953), Gas-M (*p* < 0.001; d = 2.496), VL (*p* < 0.001; d = 1.964), VM (*p* < 0.001; d = 3.092), RF (*p* < 0.001; d = 2.016), BF (*p* < 0.001; d = 2.615), ST (*p* < 0.001; d = 2.444), and Glut-M (*p* < 0.001; d = 2.571) activities during the loading phase. Pair-wise comparisons demonstrated greater TA, Gas-M, VL, VM, RF, BF, ST and Glut-M activities in the PF compared with the WPF group during the loading phase. Significant main effects for “condition” were identified for TA (*p* = 0.001; d = 2.163), Gas-M (*p* < 0.001; d = 2.599), VL (*p* = 0.001; d = 3.483), VM (*p* < 0.001; d = 3.055), RF (*p* < 0.001; d = 3.419), BF (*p* < 0.001; d = 3.137), ST (*p* = 0.001; d = 3.207), and Glut-M (*p* = 0.001; d = 3.289) activities during the loading phase. Pair-wise comparisons showed lower TA, Gas-M, VL, VM, RF, BF, ST and Glut-M activities when running with FOs compared to without during the loading phase. Significant group-by-condition interactions were found for Gas-M (*p* = 0.036; d = 0.978) and VM (*p* = 0.002; d = 1.543) activities during the loading phase. Both groups exhibited lower activities in the Gas-M (WPF: *p* = 0.005, d = 1.608; PF: *p* = 0.001, d = 2.430) and VM (WPF: *p* < 0.001, d = 2.532; PF: *p* < 0.001, d = 2.503) with the PF group showing larger Gas-M and VM when running with FOs (Table 4).

**Table 4:** Means and standard deviations of muscle activity during the loading phase [% maximum voluntary isometric contraction (MVIC)] when running in PF group compared with WPF group (with and without FO)

Muscles	WPF group				PF group				Main effect of group (eta squared)	Main effect of condition (eta squared)	Group-by-condition interaction (eta squared)
	Pre	Post	∆%	95%CI	Pre	Post	∆%	95%CI			
***TA***	105.35 ± 31.29	61.35 ± 18.08	-41.76	21.79, 66.21	148.61 ± 38.37	109.91 ± 36.2	-26.04	8.56, 68.84	< 0.001 (1.953)*	0.001 (2.163)*	0.754 (0.142)
***Gas-M***	105.77 ± 44.60	60.84 ± 11.26	-42.47	16.84, 73.02	193.86 ± 58.20	94.63 ± 23.47	-51.18	52.16, 146.30	< 0.001 (2.496)*	< 0.001 (2.599)*	0.036 (0.978)*
***VL***	99.53 ± 39.85	41.83 ± 8.53	-57.97	19.41, 34.72	147.17 ± 34.44	68.8 ± 19.83	-53.25	46.89, 117.69	< 0.001 (1.964)*	0.001 (3.483)*	0.238 (0.532)
***VM***	74.33 ± 13.32	47.26 ± 8.07	-36.41	31.52, 83.87	153.78 ± 45.73	71.49 ± 20.04	-53.51	51.24, 105.51	< 0.001 (3.092)*	< 0.001 (3.055)*	0.002 (1.543)*
***RF***	84.22 ± 35.09	38.22 ± 7.51	-54.61	23.36, 68.62	127.26 ± 27.44	61.26 ± 17.18	-51.86	44.02, 87.98	< 0.001 (2.016)*	< 0.001 (3.419)*	0.177 (0.610)
***BF***	76.47 ± 36.03	35.08 ± 6.64	-54.12	15.03, 60.21	122.26 ± 19.04	59.93 ± 17.92	-50.98	44.51, 91.01	< 0.001 (2.615)*	< 0.001 (3.137)*	0.052 (0.899)
***ST***	72.18 ± 35.52	34.56 ± 6.16	-52.11	18.29, 64.50	124.07 ± 21.98	56.31 ± 16.99	-54.61	41.63, 83.02	< 0.001 (2.444)*	**0.001** (3.207)*	0.153 (0.648)
***Glut-M***	72.53 ± 35.76	32.47 ± 5.54	-55.23	17.56, 62.57	122.87 ± 21.31	54.65 ± 16.13	-55.52	45.85, 90.57	< 0.001 (2.571)*	0.001 (3.289)*	0.063 (0.857)

Notes: WPF, without pronatd feet; PF, pronated feet; TA, tibialis anterior; Gas-M, gastrocnemius medialis; BF, biceps femoris; ST, semitendinosus; VL, vastus lateralis; VM, vastus medialis; RF, rectus femoris; Glut-M, gluteus medius; 95% CI, confidence interval refers to the confidence interval of the difference between with and without FO. Significant results were denoted in bold

We observed significant main effects of “group” for VL (*p* = 0.044; d = 0.934) activation during the mid-stance phase. The pair-wise comparison demonstrated lower VL activities in PF compared to WPF individuals (Table 5). The analysis further indicated significant main effects of “condition” for TA activities (*p* = 0.043; d = 0.940). The pair-wise comparison demonstrated greater TA activities during the mid-stance phase of running with FOs. No significant group-by-condition interactions were found for muscle activities during the mid-stance phase (Table 5, *p* > 0.05).

**Table 5:** Means and standard deviations for muscle activations during the mid-stance phase (% maximum voluntary isometric contraction [MVIC]) when running in PF compared with WPF individuals (with and without FO)

Muscles	WPF group				PF group				Main effect of group (eta squared)	Main effect of condition (eta squared)	Group-by-condition interaction (eta squared)
	Pre	Post	∆%	95%CI	Pre	Post	∆%	95%CI			
***TA***	81.38 ± 35.86	91.13 ± 46.49	11.98	-41.10, 21.61	70.19 ± 33.8	111.77 ± 40.23	59.23	-84.98, 1.82	0.683 (0.180)	0.043 (0.940)*	0.196 (0.582)
***Gas-M***	85.33 ± 34.41	86.36 ± 32.31	1.20	-23.59, 21.55	79.84 ± 35.2	82.81 ± 23.11	3.71	-32.49, 26.54	0.666 (0.191)	0.812 (0.110)	0.907 (0.063)
***VL***	85.37 ± 32.64	83.44 ± 33.99	-2.26	-25.46, 32.11	72.63 ± 36.25	64.15 ± 21.67	-11.67	-15.67, 37.66	0.044 (0.934)*	0.431 (0.352)	0.672 (0.191)
***VM***	80.94 ± 30.56	77.61 ± 32.37	-4.11	-27.80, 31.66	65.99 ± 35.76	54.99 ± 17.63	-16.66	-21.41, 38.38	0.096 (0.763)	0.591 (0.238)	0.735 (0.155)
***RF***	73.19 ± 27.86	66.45 ± 25.49	-9.20	-17.74, 31.23	61.82 ± 36.59	49.53 ± 14.71	-19.88	-14.20, 38.79	0.090 (0.773)	0.255 (0.510)	0.737 (0.155)
***BF***	77.51 ± 23.9	60.43 ± 22.95	-22.03	-4.39, 35.99	59.44 ± 37.31	48.93 ± 12.58	-17.68	-15.12, 33.94	0.118 (0.710)	0.091 (0.773)	0.658 (0.201)
***ST***	73.78 ± 24.38	57.98 ± 20.13	-21.41	-4.04, 38.20	57.96 ± 37.81	48.55 ± 11.79	-16.23	-14.45, 35.49	0.071 (0.830)	0.074 (0.820)	0.660 (0.191)
***Glut-M***	73.46 ± 25.67	57.39 ± 19.68	-21.87	-5.31, 37.45	60.37 ± 35.8	46.65 ± 11.4	-22.72	-9.77, 37.23	0.124 (0.700)	0.050 (0.908)	0.872 (0.063)

Notes: WPF, without pronatd feet; PF, pronated feet; TA, tibialis anterior; Gas-M, gastrocnemius medialis; BF, biceps femoris; ST, semitendinosus; VL, vastus lateralis; VM, vastus medialis; RF, rectus femoris; Glut-M, gluteus medius; 95% CI, confidence interval refers to the confidence interval of the difference between with and without FO. Significant results were denoted in bold

We found significant “group” effects for TA (*p* = 0.001; d = 1.816), and Gas-M (*p* = 0.038; d = 0.965) activities during the push-off phase of running. Pair-wise comparisons demonstrated greater TA and Gas-M activities in the PF group. No significant main effects of “condition” were found for muscle activities during the push-off phase (Table 6, *p* > 0.05). Significant group-by-condition interactions were observed for VL (*p* = 0.045; d = 0.931), RF (*p* = 0.047; d = 0.918), BF (*p* = 0.038; d = 0.969), ST (*p* = 0.043; d = 0.940), and Glut-M (*p* = 0.012; d = 1.207) activities during the push-off phase. Post-hoc analyses indicated greater VM, RF, BF, ST, and Glut-M activities in the WPF group (but not the PF group) during the push-off phase while running with FOs (Table 6).

**Table 6:** Means and standard deviations for muscle activations during the push-off phase (% maximum voluntary isometric contraction [MVIC]) when running in PF compared with WPF individuals (with and without FO)

Muscles	WPF group				PF group				Main effect of group (eta squared)	Main effect of condition (eta squared)	Group-by-condition interaction (eta squared)
	Pre	Post	∆%	95%CI	Pre	Post	∆%	95%CI			
***TA***	56.93 ± 24.23	63.97 ± 25.95	12.36	-28.86, 14.80	100.56 ± 35.26	98.01 ± 22.01	-2.53	-15.56, 20.66	< 0.001 (1.816)*	0.733 (0.155)	0.468 (0.320)
***Gas-M***	59.99 ± 22.51	76.78 ± 31.19	27.98	-42.10, 8.53	90.18 ± 34.88	86.31 ± 20.97	-4.29	-16.74, 24.49	0.038 (0.965)*	0.397 (0.375)	0.181 (0.602)
***VL***	52.24 ± 21.35	73.78 ± 37.63	41.23	-59.17, -2.84	78.56 ± 35.87	69.18 ± 15.09	-11.93	-19.17, 29.56	0.195 (0.586)	0.144 (0.663)	0.045 (0.931)*
***VM***	45.79 ± 20.51	76.8 ± 35.65	67.72	-50.34, 7.27	75.86 ± 37.29	70.66 ± 20.32	-6.85	-14.22, 32.98	0.226 (0.544)	0.483 (0.314)	0.084 (0.793)
***RF***	43.83 ± 20.7	67.33 ± 28.92	53.61	-48.23, 1.23	70.34 ± 36.66	61.18 ± 15.76	-13.02	-14.40, 32.71	0.216 (0.557)	0.365 (0.403)	0.047 (0.918)*
***BF***	41.72 ± 20.84	63.44 ± 23.86	52.06	-40.61, 0.03	68.21 ± 39.34	58.51 ± 14.83	-14.22	-13.48, 38.08	0.113 (0.721)	0.592 (0.238)	0.038 (0.969)*
***ST***	40.12 ± 21.04	60.41 ± 22.17	50.57	-43.11, -0.32	69.23 ± 39.33	56.93 ± 13.18	-17.76	-14.75, 34.16	0.198 (0.582)	0.420 (0.358)	0.043 (0.940)*
***Glut-M***	38.56 ± 20.95	58.78 ± 19.49	52.43	-38.73, -1.71	72.74 ± 38.96	55.39 ± 12.76	-23.85	-6.79, 41.50	0.062 (0.860)	0.835 (0.090)	0.012 (1.207)*

Notes: WPF, without pronatd feet; PF, pronated feet; tibialis anterior; Gas-M, gastrocnemius medialis; BF, biceps femoris; ST, semitendinosus; VL, vastus lateralis; VM, vastus medialis; RF, rectus femoris; Glut-M, gluteus medius; 95% CI, confidence interval refers to the confidence interval of the difference between with and without FO. Significant results were denoted in bold

## Discussion

The findings of this study indicate that the application of double-density FOs has the potential to modify both, GRFs and muscle activation patterns particularly in PF individuals during running at constant speed. More specifically, the application of double- density FOs resulted in higher GRFs (e.g., peak F_z_) during running in PF individuals. Irrespective of PF, lower muscle activities (e.g., vastus medialis) were found when running with double- density FOs.

### Ground reaction forces

Our results demonstrated lower peak negative free moments in individuals with but not without PF. It has previously been postulated that PF may alter the positioning of the tibia and femur compared to the normal lower limb alignment [42]. This altered alignment pattern may lead to changes in free moments, which represent the vertical moment exerted at the center of pressure and have been associated with lower limb torsional stress and tibial stress fractures in runners [46, 47]. The lower peak negative free moment is possibly a compensatory mechanism in the PF group indicating a potential decrease in the rotational forces acting at the knee and hip joints during running. Our results did not demonstrate any significant main effect of FO on peak free moment values. Previously, Jafarnezhadgero et al. observed that dual-stiffness spike distance running shoes, compared to single-stiffness models, resulted in significantly lower peak negative free moments in runners [1]. This discrepancy may be due to differences in study methodology (e.g., double- density FO versus dual stiffness spike running shoes).

Previously, researchers have identified peak vertical impact GRFs, time-to-peak for GRFs, and free moments as predictors of RRIs [48]. We found greater F_yPO_, time-to-peak for F_xPO_, time-to-peak for F_yHC_, and time-to-peak for F_zHC_ in the PF group than in the WPF group. Temporal parameters, such as time-to-peak for F_xPO_, time-to-peak for F_yHC_, and time-to-peak for F_zHC_ reflect the timing of force application during running [34]. Mafi et al. [49] found consistent evidence and argued that the increase in time-to-peak for F_zHC_ could be associated to lower rate of injuries. Moreover, the observed increase in force metrics such as F_yPO_ during running may reflect the runner’s ability to effectively propel their body. This force is generated primarily through the activation of the posterior chain muscles, including the glutes and hamstrings, which play a pivotal role in thrusting the body forward [50]. Adequate muscle activation lead to more powerful push-offs, enabling greater acceleration and speed. This physiological adaptation is crucial for athletes aiming to improve their performance metrics [46].

Our findings demonstrated lower Fz_HC_, Fz_PO_, time-to-peak for Fx_PO_, and time-to-peak for Fz_HC_ during running with FO, and lower time-to-peak for F_xPO_, time-to-peak for Fy_HC_ and time-to-peak for F_zHC_ in the PF group (but not in the WPF group) during running with FO. We observed lower F_zHC_, and F_zPO_ force metrics during running with FOs which suggests that FOs may help to lower the overall load experienced at the lower extremities, especially in the vertical plane [51].This drop in peak forces could be particularly beneficial for individuals with adverse health conditions such as patellofemoral pain or other musculoskeletal disorders, as it may alleviate stress on the joints and the surrounding tissues [52]. Moreover, the application of double density FO appears to have an impact on the loading phase during running and may thus be a potential candidate that explains the observed findings of this study in the PF versus control group. The reduced time-to-peak for forces during the push-off phase in medial direction (time-to-peak for F_xPO_), heel contact phase in anterior-posterior (time-to-peak for Fy_HC_) and vertical (time-to-peak for F_zHC_) directions may suggest that adults adjust their motor programs during motion control. This may allow to program and thus tailor a stored motor program to meet the specific demands when using FOs [53, 54]. Specifically, our findings revealed that the application of FOs significantly modify selected force metrics and temporal parameters during running, which may be exploited for rehabilitation and athletic performance enhancement purposes [55].

### Muscle activations

This study revealed higher TA muscle activity in individuals with PF which can be interpreted as a neuromuscular adaptation to compensate for excessive PF. From a clinical perspective, the foot arch collapse appears to be a biomechanical factor that increases plantar fascia tension [56] and the length-tension relationship of ankle invertor muscles such as the TA. Excessive foot pronation has often been associated with increased TA activation, as this muscle stabilizes the foot and prevents over pronation. The TA functions to control the foot’s descent and prevents the foot from excessively rolling inward [57]. Abnormal foot pronation can alter the activation patterns of key lower limb muscles and may thus increase the risk of sustaining injuries [58]. Recent studies report that individuals with PF experience higher loads on the VM than those with normal feet due to weakened plantar flexor muscles. The VM muscles play a key role in stabilizing and orienting the patella by drawing it along a slant line as it passes through the intercondylar areas of the femur [59]. We speculate that this condition could be the reason for the higher VM muscle activation in individuals with PF [60]. The increased Glut-M muscle activity might be associated with reduced foot pronation, leading to an adapted lower limb alignment and Glut-M muscle activation. A study examining the effects of excessive foot pronation found that individuals with PF had increased Glut-M activity which has been interpreted as a compensatory mechanism to stabilize the pelvis and knee [42]. The activation of the quadriceps and hamstring muscles during the loading phase of running is indicative of their role in generating the necessary force to support the body’s mass and maintain stability while in dynamic motion. This synergistic muscle activation not only enhances performance but also significantly reduces the risk of injury. Furthermore, the interplay between these muscle groups highlights the importance of neuromuscular control and the individual’s running mechanics. Proper activation patterns contribute to efficient movement and optimal running control, which are crucial to improve their overall performance.

More specifically, we found lower TA, Gas-M, VL, VM, RF, BF, ST and Glut-M activities in the FO condition during the loading phase. Double density FO similar to motion control footwear adopts different medial and lateral midsole hardness that allows more time during the loading phase of landing/running. Thus, the foot stabilizing muscles can be activated for longer periods with less intensity. This design feature may result in an overall decrease in muscle activity [61]. The application of double density FOs resulted in a reduced external eversion moment during the early stance phase. In accordance with Newton’s third law, less force might be required from TA and tibialis posterior muscles to resist eversion force This could facilitate the healing process when treating TA and tibialis posterior tendon dysfunction [62]. Recent research has yielded insightful findings regarding the influence of FOs on muscle activity during the loading phase. The lower muscle activity may suggest that FOs may provide joint stability in PF individuals due to their inherent characteristics (e.g., double density) during the loading phase. In addition, the lower muscular activities when wearing FOs may indicate a shift in loading distribution, potentially alleviating strain on specific muscle groups, thus leading to a different biomechanical response [29]. Specifically, the pronounced decrease in Gas-M activity, particularly within the PF group, warrants further investigation with regards to the applications of FOs on lower limb mechanics and injury prevention strategies.

Findings from this study indicated lower VL activities in PF individuals during the mid-stance phase of running. In addition, higher TA activities were found in the FO condition only during the mid-stance phase of running. Recent findings revealed that adults with PF exhibited significantly lower VL activities compared to WPF individuals [60]. This disparity in muscle activation may indicate an underlying dysfunction or adaptation in the PF group, which may have implications for joint stability during dynamic activities such as running.

Moreover, this study revealed higher TA activity when running with FOs during the mid-stance phase. Elevated TA activation when running with FOs may suggest that FOs could have the potential to improve neuromuscular control and thus the loading patterns in lower extremity muscles [42]. The TA muscle (the primary invertor of the foot) acts eccentrically during loading phase to generate an inversion moment that opposes the external eversion moment, and helps control rearfoot eversion [63]. More specifically, higher TA activities appear to better stabilize the ankle and foot, which is a practically and clinically relevant finding in athletic and rehabilitative settings [63].

We observed higher VM, RF, BF, ST, and Glut-M activities in the WPF group (but not in the PF group) during the push-off phase while running with FO. Higher TA and Glut-M activities in the PF cohort suggest a compensatory mechanism in response to the altered biomechanics often associated with the PF syndrome [42]. This compensatory activation may contribute to the pain and dysfunction typically observed in these individuals, as the reliance on these muscles could indicate an adaptation to stabilize the knee joint under compromised conditions. In contrast, the WPF group demonstrated higher muscle activations in the VM, RF, BF, ST, and Glut-M during the push-off phase when utilizing functional orthotics. Higher VM, RF, BF, ST, and Glut-M activities may lead to better power transmission and overall running efficiency/economy [64]. The application of FOs appears to facilitate muscle activation which improves performance, particularly in PF individuals.

### Study limitations

We acknowledge that the study cohort was relatively small. However, the a priori power analysis indicated sufficient statistical power to detect differences and interactions between groups and conditions. Further research is needed to assess the effects of different running speeds and the slope level. Moreover, only adult males were enrolled in this study. Therefore, it is not possible to generalize our findings to other populations, i.e. older adults, youth or females. Future research should examine whether similar effects can be found in these populations. Lastly, the inclusion of kinematic methods may additionally provide information besides the assessment of kinetic and electromyographic data.

## Conclusions

The current findings suggest that the application of double- density FOs resulted in higher GRFs (e.g., peak F_z_) during running in PF individuals. Irrespective of PF, the wearing of double density FOs resulted in lower muscle activation (e.g., vastus medialis). Accordingly, FOs can be applied as therapeutic means to treat runners, particularly with PF.

## Electronic supplementary material

Below is the link to the electronic supplementary material.

Supplementary Material 1

Supplementary Material 2

## Data Availability

The datasets used and/or analysed during the current study are available from the corresponding author on reasonable request.

## Abbreviations

PF: Pronated feet
BMI: Body-mass-index
WPF: Without pronated feet
GRFs: Ground reaction forces
EMGs: Surface electromyography
RRIs: Running-related injuries
FOs: Foot orthoses
EVA: Ethylene-vinyl acetate
BW: Body weight
x: Medio-lateral direction
y: Anterior-posterior direction
z: Vertical direction
F_zHC_: Peak vertical ground reaction force during heel contact
F_zPO_: Peak vertical ground reaction force during the push-off phase
F_yHC_: Braking force
F_yPO_: Propulsion force
F_xHC_: Peak lateral ground reaction force during heel contact
F_xPO_: Peak medial ground reaction force during the push-off phase
FM: Free moment
TTP: Time-to-peak
95% CI: Confidence interval
COP: Center of pressure
TA: Tibialis anterior
Gas-M: Gastrocnemius medialis
BF: Biceps femoris
ST: Semitendinosus
VL: Vastus lateralis
VM: Vastus medialis
RF: Rectus femoris
Glut-M: Gluteus medius
SENIAM: Surface electromyography for non-invasive assessment of muscles
RMS: Root mean square
MVIC: Maximum voluntary isometric contraction
SD: Standard deviations
