# Supplementary material for: Effect of double- density foot orthoses on ground reaction forces and lower limb muscle activities during running in adults with and without pronated feet
Source: BMC Sports Sci Med Rehabil. 2025 Mar 21;17:54. doi: 10.1186/s13102-025-01095-5 (PMC11927312; doi:10.1186/s13102-025-01095-5)
Supplement: Supplementary file 2 — Supplementary Material 2 [file 13102_2025_1095_MOESM2_ESM.doc]

**Allocation**

**Analysis**

**Enrollment**

Assessed for eligibility (N= 40)

Excluded (n= 0)

Analysed (n= 20)
♦ Excluded from analysis (n= 0)

Allocated to pronated feet group (n= 20)

♦ Received allocated intervention (n= 20)

♦ Did not receive allocated intervention (give reasons) (n= 0)

Allocated to healthy group (n= 20)

♦ Received allocated intervention (n= 20)

♦ Did not receive allocated intervention (give reasons) (n= 0)

Analysed (n= 20)
♦ Excluded from analysis (n= 0)

Allocation (n= 40)
